# Comment on Häuser et al.: Medical use of opioids in Europe—Methodological concerns about data from the *International Narcotics Control Board*

**DOI:** 10.1002/ejp.1919

**Published:** 2022-02-09

**Authors:** Emmanuel Bäckryd, Markus Heilig, Mikael Hoffmann

**Affiliations:** ^1^ Department of Health, Medicine and Caring Sciences Pain and Rehabilitation Centre Linköping University Linköping Sweden; ^2^ Center for Social and Affective Neuroscience, Department of Biomedical and Clinical Sciences (BKV) Linköping University Linköping Sweden; ^3^ The NEPI foundation and Unit of Health Care Analysis Department of Health, Medicine and Caring Sciences Linköping University Linköping Sweden

Although opioids are necessary medicines, overprescribing is a serious issue, and it is, therefore, important to learn from the US opioid crisis. In a recent paper in your journal, it was concluded that ‘Europe as a whole is not facing an opioid crisis’ (Häuser et al., 2021). Based on data from the Swedish Medical Products Agency and on recent peer‐reviewed papers (Bäckryd et al., 2017, 2021; Jarlbaek, 2019), we concur at least concerning the Scandinavian countries. However, it is with great surprise that we note that Häuser et al, relaying Bosetti et al. (2019), put such a heavy emphasis on data from the *International Narcotics Control Board* (INCB). Indeed, Table 1 in the Häuser paper is based solely on INCB data. This is problematic in many ways. For one thing, the table gives the impression that there is, indeed, an opioid crisis in Europe, and hence there is a deep incongruence between the conclusion of the study and the way results are presented in the paper's only table. We acknowledge that the authors recognize that INCB data have limitations, but we think that those limitations are understated. Opioid availability, as expressed by INCB statistics, does not reflect medical opioid use. There are deep methodological problems with INCB opioid statistics, and we have previously shown (Bäckryd et al., 2021) that INCB data are markedly inconsistent with actual sales from Scandinavia, calling the reliability of the data into question. At least in a Scandinavian context, INCB statistics are deeply flawed, for example, by over‐representing the volume of fentanyl, by under‐reporting codeine, and by not including tramadol. These are not marginal divergences. Instead, the discrepancies between sales statistics and INCB data are staggering, in terms of both trends and volumes (Bäckryd et al., 2021). Frankly, based on these findings, we question the use of INCB data when analysing medical use of opioids in a scientific journal. Hence, although we agree with Häuser et al in their statement that there is no clear indication that Europe is facing an opioid crisis as the one described in the United States, and although we applaud the endeavour to study this important question, we think that their use of INCB statistics is highly problematic. We understand that there might be a paucity of data in many countries, but sometimes acknowledging a lack of data is better than reproducing doubtful data.

## References

[ejp1919-bib-0001] Bäckryd, E. , Heilig, M. , & Hoffmann, M. (2017). Opioid prescription changes in Sweden 2000–2015 [Article in Swedish]. Läkartidningen, 114, EFUE. 28485763

[ejp1919-bib-0002] Bäckryd, E. , Heilig, M. , & Hoffmann, M. (2021). Opioid availability statistics from the International Narcotics Control Board do not reflect the medical use of opioids: Comparison with sales data from Scandinavia. Scandinavian Journal of Pain, 21(4), 696–706. 10.1515/sjpain-2021-0023 34315195

[ejp1919-bib-0003] Bosetti, C. , Santucci, C. , Radrezza, S. , Erthal, J. , Berterame, S. , & Corli, O. (2019). Trends in the consumption of opioids for the treatment of severe pain in Europe, 1990–2016. European Journal of Pain, 23(4), 697–707. 10.1002/ejp.1337 30407692

[ejp1919-bib-0004] Häuser, W. , Buchser, E. , Finn, D. P. , Dom, G. , Fors, E. , Heiskanen, T. , Jarlbaek, L. , Knaggs, R. D. , Kosek, E. , Krcevski‐Škvarč, N. , Pakkonen, K. , Perrot, S. , Trouvin, A.‐P. , & Morlion, B. (2021). Is Europe also facing an opioid crisis?—A survey of European Pain Federation chapters. European Journal of Pain, 25(8), 1760–1769. 10.1002/ejp.1786 33960569

[ejp1919-bib-0005] Jarlbaek, L. (2019). Opioid prescribing habits differ between Denmark, Sweden and Norway—And they change over time. Scandinavian Journal of Pain, 19(3), 491–499. 10.1515/sjpain-2018-0342 30817310

